# Applications of RNA interference high-throughput screening technology in cancer biology and virology

**DOI:** 10.1007/s13238-014-0076-6

**Published:** 2014-06-22

**Authors:** Shan Gao, Chen Yang, Shan Jiang, Xiao-Ning Xu, Xin Lu, You-Wen He, Annie Cheung, Hui Wang

**Affiliations:** 1 Department of Oncology, John Radcliffe Hospital, Weatherall Institute of Molecular Medicine, University of Oxford, Oxford, OX3 9DS UK; 2 Department of Molecular Genetics and Microbiology, Stony Brook University, Stony Brook, NY 11794 USA; 3 Department of Immunology, Duke University, Durham, NC 27708 USA; 4 Department of Immunology, Chelsea & Westminster Hospital, Imperial College of London, 369 Fulham Road, London, SW10 9NH UK; 5 Nuffield Department of Clinical Medicine, Ludwig Institute for Cancer Research Oxford Branch, University of Oxford, Old Road Campus Research Building, Oxford, OX3 7DQ UK; 6 Department of Pathology, Queen Mary Hospital, University of Hong Kong, Pokfulam Road, Hong Kong, China; 7 Natural Environment Research Council, Centre for Ecology & Hydrology, Benson Lane, Wallingford, Oxfordshire OX10 8BB UK; 8 Department of Zoology, University of Oxford, South Parks Road, Oxford, OX1 3PS UK

**Keywords:** RNA interference (RNAi), short interfering RNA (siRNA), short hairpin RNA (shRNA), high-throughput screening, cancer, virology

## Abstract

RNA interference (RNAi) is an ancient intra-cellular mechanism that regulates gene expression and cell function. Large-scale gene silencing using RNAi high-throughput screening (HTS) has opened an exciting frontier to systematically study gene function in mammalian cells. This approach enables researchers to identify gene function in a given biological context and will provide considerable novel insight. Here, we review RNAi HTS strategies and applications using case studies in cancer biology and virology.

## Introduction

The systematic gene networks remain challenging, after many genomes from different species have been sequenced within the last two decades (Adams et al., 2000; Lander et al., 2001; Waterston et al., 2002; Yu et al., 2002). Although the functions of an increasing number of gene products have been revealed, a major challenge in the post-genomic era remains to clarify the precise roles of these genes in specific biological processes and ultimately to develop new strategies to fight diseases. RNA interference (RNAi) technology (Fire et al., 1998) is a powerful tool to study gene function by silencing transcription. Significant progress has been made using RNAi technology in the investigation of the molecular basis of cancer development. Such studies have identified several novel oncogenes, such as *sequence similarity 83, member B* (*FAM83B*) in breast cancer (Cipriano et al., 2012), *LIM-homeodomain-containing transcription factor 1B* (*Lmx1b*), *p21-activated kinase 4*, *inhibitor of apoptosis-stimulating protein of p53* (*iASPP*), and stem cell transcription factor *Nanog* in ovarian cancer (Siu et al., 2010; Jiang et al., 2011; He et al., 2013; Siu et al., 2013), and *abnormal spindle homologue, microcephaly associated* (*ASPM*) in glioblastoma (Horvath et al., 2006). Significant progress has also been made in the field of host immune responses against virus infections using RNAi technology. Indeed, many novel genes involved in virus entry and replication in the host have been uncovered (Brass et al., 2008; Brass et al., 2009).

With such technological advances, it is now feasible to interrogate phenotypes associated with the loss-of-function of many genes in mammalian cell culture systems. Furthermore, it becomes possible to construct genome-wide RNAi libraries that systematically target every individual gene in a given genome to perform high-throughput screening (HTS) of specific phenotypes of interests (Silva et al., 2008). The combination of RNAi and HTS in cell culture systems, known as cell-based RNAi HTS, is a new and exciting frontier in basic and applied biology. Inevitably, such a powerful methodology has led to significant progress in many areas of cancer biology, i.e., the crosstalk of biological signaling and the identification and validation of cancer therapeutic targets (Moffat and Sabatini, 2006; Iorns et al., 2007).

In this review, we provide an overview of RNAi and RNAi HTS in cell systems. Different technical strategies are described using case studies as examples. Finally, discussions are made in the context of the existing problems with these screens.

## RNAi

RNAi is an evolutionarily conserved mechanism that regulates gene expression in eukaryotic cells. In this process, double-stranded RNAs (dsRNAs) can suppress the expression of target genes in a homology-dependent manner (Hannon, 2002; Almeida and Allshire, 2005). The effectors of RNAi are short interfering RNAs (siRNAs) produced from long dsRNA substrates by an RNAse III enzyme called Dicer. These Dicer products are 20- to 25-bp-long dsRNAs with a characteristic 2-nt overhang at the 3′-end (Fire et al., 1998; Bernstein et al., 2001). The siRNA duplexes are incorporated into the RNA-induced silencing complex (RISC) containing a core Argonaute (AGO) protein that destroys a “passenger strand” and keeps the “guiding strand” that hybridizes to the target mRNA by complementary homology. Such a homology-based association allows the AGO to degrade the mRNA transcript, or to inhibit the mRNA translation, resulting in post-transcriptional gene silencing (Martinez et al., 2002; Hock and Meister, 2008).

In mammalian cells, long dsRNAs induce interferon responses, which are the first line of defense against viral infections, and subsequently lead to a global shutdown of protein synthesis (Reynolds et al., 2006). However, vectors expressing short hairpin RNAs (shRNAs) and/or synthetic siRNAs designed to mimic endogenous 21-nt siRNAs can be manually introduced into mammalian cells to avoid the interferon response, thus mediating gene silencing without significant adverse effects (Elbashir et al., 2001; Kim and Rossi, 2007). With the availability of completely sequenced genomes, siRNA or vector-based shRNA libraries can be constructed with specific designs to maximize the probability of potent target gene silencing and to minimize the risk of off-target effects (Huesken et al., 2005; Turner et al., 2008). To date, RNAi has been successfully developed to become a powerful experimental tool (Huang et al., 2013)

## RNAi HTS

Traditionally, functional genetic studies have been performed by so called forward genetics, in which random gene mutations are generated by induction (e.g., radiation, chemical treatments, and/or insertional mutagenesis), and then mutants with specific phenotypes are identified by breeding and segregation processes (Gao et al., 2008; Lawson and Wolfe, 2011). However, such procedures are time-consuming and not easily applicable to mammalian cell systems. Conversely, reverse genetic strategies focus on the mutants of a gene of interest by observing the phenotype so as to determine the gene function (Wang et al., 2011). Traditional gene knockouts, either in cell systems or living organisms, are expensive, time-consuming, and unsuitable for genome-scale screens. Using RNAi technology, a gene that is required for a certain function can be silenced by the introduction of siRNAs, and the corresponding phenotype can be determined by appropriate assays. This fascinating link between phenotype and RNAi-mediated gene silencing has promoted rapidly growing applications of RNAi in functional genomics, signal transduction, and drug target discovery. As such, RNAi represents a major technological advance for performing large-scale screenings in cell culture systems (Echeverri and Perrimon, 2006). HTS is a procedure that supports large-scale experiments, allowing a researcher to quickly conduct thousands or millions of chemical screens or genetic tests simultaneously (Krausz, 2007).

## RNAi HTS methods

**Figure 1:**
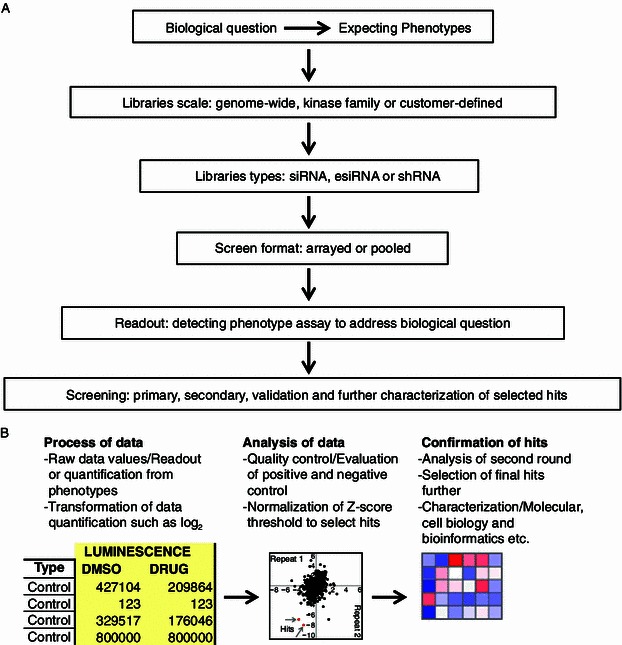
Flowchart of RNAi high-throughput screening. (A) The design and steps of a high-throughput screen. The choice of screen libraries such as scale, type, or format can be determined based on biological questions or the phenotypic assays developed. (B) Data analysis (Gao et al., unpublished data). Raw data can be analyzed according to your choice of statistical methods. Every experiment is subject to quality control (QC). If QC is passed, the primary hits are selected for a second round of screening. The final hits can be confirmed and further characterized using molecular biology, cell biology, or bioinformatics to determine their biological meanings

In the initial stage of an RNAi HTS experiment, the purpose of the screening should be clear, such as the identification of regulators for cell proliferation, size, morphology, adhesion, death, division, or the efficacy of viral infection. These questions enable researchers to develop corresponding phenotypic assays that should be clearly defined and easily scored with interpretations in relevant biological contexts (Boutros and Ahringer, 2008). Then, one can choose the appropriate type of RNAi library to screen (i.e., either genome-wide or for a subset of gene families of interest) and develop robust and reproducible assays for identification, validation, and characterization of candidate genes (Fig. 1A). Technical details that should be considered include: i) delivery methodology of the RNAi, ii) raw data collection, iii) appropriate positive and negative controls that can be used to optimize the discrepancy between genuine signals and background noise, iv) statistical methods that can define the initial hits, v) secondary validation assays to filter the primary hits, and vi) function determination of selected hits (Fig. 1B) (Sharma and Rao, 2009).

Currently, there are two major types of RNAi libraries that are widely used by researchers: siRNA and vector-based shRNA libraries. siRNA can be chemically synthesized or generated from cDNA templates by RNase III via a technique known as esiRNA, in which gene cDNA with RNA polymerase promoter amplified by PCR is transcribed *in vitro*, and then its products is digested by RNase III to generate siRNAs that is similar to siRNAs generated *in vivo* by Dicer. These multiple silencing triggers result in higher effective gene silencing and lower off-target effects compared to single and pooled siRNAs (Yang et al., 2002; Kittler et al., 2004). Synthetic siRNA libraries are used for most situations for short-term gene silencing because these siRNA duplexes are not replicated and are progressively diluted as cells divide. Vector-based shRNA libraries are defined according to the type of viral vector used, e.g., retroviral, adenoviral, or lentiviral. Vector-based shRNA libraries are able to provide long-term and stable gene silencing because the vectors integrate into genomic DNA and are thus replicated. In particular, lentiviral-based shRNA libraries are quite useful in some cells, such as primary cells, that are difficult to transfect.

There are two distinguishable strategies used in RNAi screens. One is the array-based screen, and the other is pooled shRNA libraries coupled to next generation sequencing. In the array-based screen, both siRNA and shRNA can be used in this format. There are many factors to affect the generation of shRNAs, so it is hard to balance the concentration of every shRNA in HTS. However, the chemically synthesized siRNAs are easily to handle to titer their concentrations (Liu et al., 2010). Each gene of interest can be targeted by siRNA pools in one well, which is composed of three to six individual non-overlapping siRNAs, or by individual siRNAs in separated wells. After transfection (48–72 h), cells are divided into different groups that can be treated with different selective pressures. For example, cells are treated with or without a drug for another 3–7 days and then examined for the phenotype of interest. In the case of using siRNA pools, screening is normally conducted in duplicate or triplicate in the first round, and then the pools are rescreened using the deconvoluted individual siRNAs to confirm final hits. In the pooled format, off-target effects of siRNAs are reduced as the concentration of each individual siRNA is decreased, while the total amount of siRNA molecules targeting the same mRNA species maintains the same as in the individual screening format.

**Figure 2:**
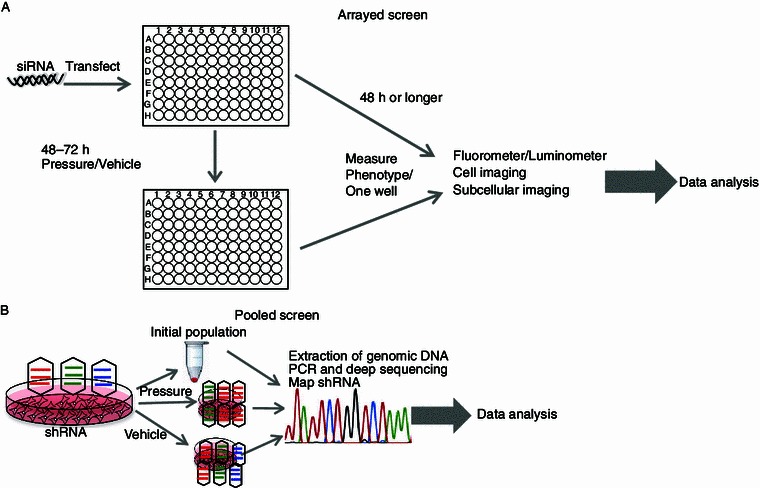
RNAi high-throughput screening approaches. (A) Arrayed screen using siRNA in a multiwell plate. siRNA can be reverse-transfected into cells for 48–72 h. Then, cells can be selected using some pressure or left for a longer time period to develop phenotypes, which can be determined using various readouts. (B) Pooled screen using pooled shRNA viral particles to infect target cells. After 48 h, cells can be divided into multiple groups. One group can be frozen as an initial population. Other groups can be treated with various pressures for some period. Then, relative enrichment of shRNAs can be analyzed using next generation sequencing

In the individual siRNA screening format, in theory, different siRNAs targeting the same mRNA species should induce an identical phenotype. In reality, these siRNAs rarely lead to the same phenotype because different siRNAs are heterogeneous in inducing Dicer-mediated degradation of the target mRNA. Furthermore, a number of phenotypes only occur while the target is depleted to a certain level. Therefore, if any two individual siRNAs can confirm the same phenotype, they are considered as the final hits. Occasionally, a high proportion of a single siRNA hit may be ignored and subsequently lead to false negatives in the same assay (Fig. 2A) (Iorns et al., 2007).

In the pooled shRNA library coupled in next generation sequencing format, shRNAs are commonly cloned into specific vector backbones with unique barcodes. This approach is widely used for pooled competitive screening, so it is often called barcode screening and shows improvements in speed and scale compared to array-based screening. A large population of cells can be infected with a pool of vector-based shRNAs. After 48–72 h of infection, cells are then spilt into three or more groups. One group is immediately frozen as the initial population, the second group (or additional groups) is treated with specific selective pressure(s) for a longer period (14 days or longer), while the last group is treated with vehicle. After the selective pressure is applied, cells are harvested from the treatment groups, and genomic DNAs are extracted from these populations. Finally integrated shRNAs are recovered using PCR amplification followed by next generation sequencing.

If a given shRNA decreases cell viability, the relative abundance of that shRNA in the vehicle group will decrease when compared to the initial population. Similarly, the shRNA target gene under pressure will affect cell viability, so its relative abundance in the pressure group will correspondingly decrease when compared to the vehicle group, which is helpful to indicate the function or network connection of the target gene (Fig. 2B) (Sims et al., 2011; Corcoran et al., 2013). Alternatively, pooled shRNA can be used to infect different cell lines. After the selection period, PCR and next generation sequencing are employed to determine which shRNA reagents are under- or over-represented in these cell lines to define targets (Silva et al., 2008).

## The application of RNAi HTS to cancer biology

The RNAi HTS approach is still at an early stage compared to many classical genetic screens, but it has already been used in a large number of studies that show some significant impact in a wide variety of fields, especially in cancer biology. Cancer cells acquire a set of mutated genes during carcinogenesis, and a vast amount of information about these mutated genes has been accumulated from whole exome sequencing and next generation sequencing (Berger et al., 2011; Banerji et al., 2012; Barbieri et al., 2012). However, that does not mean that all of these mutated genes prompt cancer development, rather than only a limited mutated genes causing the cancer (Ashworth et al., 2011). It is crucial to distinguish which mutated genes are a key driver for survival and proliferation of cancer cells. Moreover, developing potent and specific inhibitors targeting these key mutated genes represents a hot field in targeted cancer therapy, which has become an important part of many cancer treatment methods due to its precision in killing cancer cells with relatively few side effects as compared to traditional chemotherapies. However, precision often also means narrowness, which is an intrinsic drawback of targeted cancer therapy. Thus, these inhibitors often display less effective and limited activity in killing cancer cells and also allow cancer cells to develop drug resistance, one of the primary reasons for treatment failure (Guo and Wang, 2012). The major challenge and key step for cancer therapy is to identify the target that is essential for the survival and proliferation of the cancer cells, as well as biomarkers that are able to predict what types of cancers are sensitive or resistant to specific inhibitors. Therefore, RNAi HTS will be helpful in target selection and selective cancer patient treatment. Moreover, phenotypic assays to detect cell number and viability are well established. Thus, it is not surprising that RNAi HTS was applied to uncover mutant gene function or therapeutic target identification in neoplastic phenotypes in the earliest studies.

A genome-wide siRNA lethality screen has been performed to identify functional dependencies in two breast cell lines that were transformed using the same oncogene with different phenotypes. Proteasome genes were enriched among the 154 identified genes that show higher dependency in basal-like transformed cells than in myoepithelial-like transformed cells. Indeed, basal-like triple negative breast cancer (TNBC) lines are selectively sensitive to proteasome inhibitors compared to normal epithelial, luminal, and mesenchymal TNBC lines (Petrocca et al., 2013).

A genome-wide shRNA library in a pooled format has been applied to identify genes that regulate sensitivity to *RAS* mutant cancer cells in colorectal DLD-1 cells with and without a mutant form of the oncogene *KRAS*. A set of mitotic regulators, including ubiquitination, proteasome degradation of mitotic factors, and *PLK1*, increase the dependency of KRAS-mutant cells on mitotic checkpoints and progression. These KRAS-mutant cell lines are also more sensitive to treatment with a PLK1 inhibitor in both *in vivo* and xenograft models. PLK1 inhibitors (http://www.clinicaltrials.gov/ct2/results?term=PLK1&Search=Search) and siRNAs (http://www.tekmira.com/pipeline/tkm-plk1.php) are currently in clinical trials, and it will be very interesting to determine if RAS tumors display increased sensitivity in a clinical setting (Luo et al., 2009). MED12 was identified as a common determinant of drug resistance using 24,000 shRNAs targeting 8000 human genes in a lung cancer line harboring a translocation between *EML4* and the kinase *ALK*, which is sensitive to ALK inhibitors PF-02341066 (crizotinib) and NVP-TAE684. Furthermore *MED12* silencing causes resistance to various tyrosine receptor inhibitors by negatively regulating *TGF-βR2* via interaction with *TGF-βR2* (Huang et al., 2012).

Although several groups have made significant progress in the identification of key oncogenic events and cancer therapeutic targets using RNAi HTS, nearly all of these screens were conducted *in vitro*. Such *in vitro* screens result in some key and novel findings in the studies of cancer cells but are less able to recapitulate the complex interactions between tumors and their microenvironment, which is an important step toward understanding cancer cell growth in a more physiologic context, as it is not possible to design more rational treatments for cancers based on *in vitro* screens (Mbeunkui and Johann, 2009). Beronja and colleagues performed an *in vivo* genome-wide pooled lentiviral shRNA screen in normal embryonic epidermal tissue and a hyperproliferation of *Hras*^*G12V*^-induced neoplasm in mice. A number of expected and unexpected genes that regulate embryonic epidermal growth were identified by the analysis of relative shRNA abundance. After eliminating genes that are essential under both conditions and regarded as housekeeping/viability genes, there are still ~250 candidates left that were defined as oncogene-specific growth regulators. They represent genes that could be targeted in cancer without causing any ill effect on normal tissue. Among the top *Hras*^*G12V*^-dependent screen hits were the Wnt effector β-catenin and myeloid/lymphoid or mixed-lineage leukemia translocated to 6 (Mllt6). Silencing of either gene in *Hras*^*G12V*^-expressing epidermal cells diminishes the formation of *Hras*^*G12V*^-dependent squamous papillomas in mice and inhibits both the initiation and maintenance of human squamous cell carcinoma xenografts. These results demonstrate the feasibility of this *in vivo* screening approach and identify key regulators of oncogenic growth that may represent potential therapeutic targets (Beronja et al., 2013).

## The application of RNAi HTS in virology

Despite great effort in the antiviral drug development and vaccination research fields, viruses are still major threats to public health. Indeed, despite the availability of several different vaccines, influenza viruses infect up to one billion people globally, accounting for five million cases of severe disease and 250,000 to 500,000 deaths each year (Girard et al., 2005; Lambert and Fauci, 2010). In contrast, without a vaccine after 25 years of research, HIV has infected more than 70 million people to date and is responsible for 35 million deaths (WHO). Similarly, hepatitis C virus (HCV) infects 130–170 million people each year, causing acute and chronic infection (Madan et al., 2014). Dengue virus infects near 100 million people each year, resulting in half a million cases of hemorrhagic fever (Hussain and Asgari, 2014). In addition, viruses are also the major driving forces for certain types of tumors in humans. High risk types of human papillomaviruses (HPVs) are the causative agent of nearly all cervical cancers, which is one of the leading causes of mortality in women (Rositch et al., 2014). Similarly, Epstein-Barr virus (EBV), which infects 90% of adults worldwide, plays a key role in certain tumors, including Burkitt’s lymphoma, Hodgkin’s disease, and nasopharyngeal carcinomas (Pattle and Farrell, 2006).

The most effective way to prevent virus-related diseases is vaccines. However, in most cases, vaccines are not available or not always effective. Each year, the protection efficacy of trivalent inactivated influenza vaccines (TIVs) is only ~59% in the US adult population aged 18–65 years (Osterholm et al., 2012). Antiviral drugs are therefore critical in controlling viruses. The drugs currently in use mainly target virus proteins or genomes. However, it is very easy for viruses, especially RNA viruses, to develop resistance against such drugs due to the high mutation rates of the viral genomes. To that end, finding a more effective way to develop novel drugs has become a crucial research topic. It has been shown that pooled siRNAs targeting the Zaire Ebola virus (ZEBOV) key proteins including RNA polymerase L proteins, viral protein (VP) 24 and VP35 efficiently protect against ZEBOV in non-human primates, suggesting this will be useful treatment for other emerging viral infections in human (Geisbert et al., 2010).

Viruses rely on host cells to propagate. Therefore, understanding the involvement of host factors in virus infection may facilitate the discovery of potential drug candidates because host factors undergo much less mutagenic pressure than viral proteins and may have a universal function during different virus infections. With the development of HTS RNAi screens, it became possible to examine genome-wide interactions between viruses and host factors.

**Figure 3:**
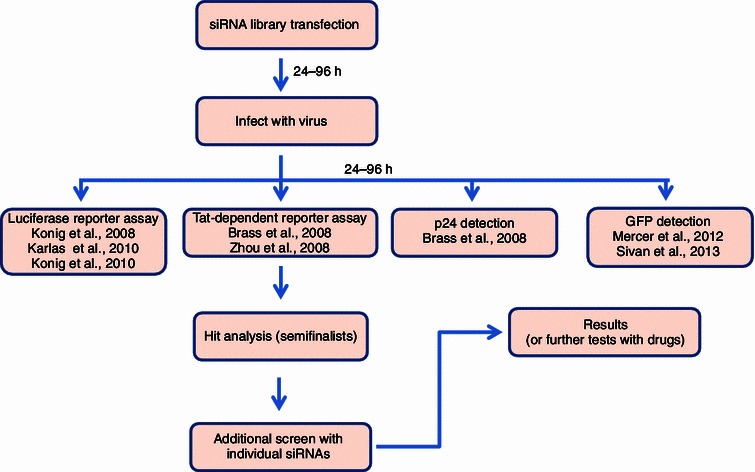
Work flow of genome wide siRNA screen for viral related host factor. Cells are initially transfected with different siRNA libraries, and then infected with viruses. Different assays can be utilized to detect screen results, including luciferase reporter assay, Tat-dependent reporter assay, p24 detection, as well as GFP detection. After first round of screen, hit analysis is performed and the candidates are subject to additional screen with individual siRNAs

The Drosophila C virus (DCV), a picornavirus, was the first virus to undergo genome-wide RNAi screening to identify novel host factors required for internal ribosome entry site (IRES)-dependent viral translation (Cherry et al., 2005). The first virus in a mammalian system that was screened by genome wide RNAi is HCV in 2007. In an effort to discover novel druggable targets against HCV, a library of siRNAs targeting 4,000 human genes was used to identify genes that regulate HCV replication in Huh7-derived EN5-3 cells harboring an HCV subgenomic replicon (Fig. 3). Nine cellular genes, including members of the tumor necrosis factor/lymphotoxin signaling pathway, were identified (Table 1). Silencing these genes leads to inhibition of HCV replication, and the level of siRNA silencing of these host genes correlates well with the inhibition of HCV (Ng et al., 2007).

**Table 1:** Genome-wide siRNA screen for host factors related to virus infections

Virus	Group	Cells	Gene targeted	siRNAs per gene	Host factors identified	
HCV	Ng et al. (2007)	EN5-3 (Huh7)	4000	4	9	
HIV	Brass et al. (2008)	TZM-bl	21,121	4	273	3 overlap MED6, MED7, RelA.
	Zhou et al. (2008)	P4/R5 (HeLa)	19,709	3	311	
	Konig et al. (2008)	HEK-293T	19,628	6	295	
Influenza	Hao et al. (2008)	DL1	13,071	4	100	3 overlap ARCN1, ATP6AP1, COPG
	Brass et al. (2009)	U2OS	17,877	4	250	
	Konig et al. (2010)	A549	19,000	4	295	
	Karlas et al. (2010)	A549	22,843	4	168	
VACV	Mercer et al. (2012)	HeLa MZ	6979	3	188	23 overlap
	Sivan et al. (2013)	HeLa	21,566	4	500	

In 2008, three groups simultaneously reported genome-wide RNAi HTS for host factors required for HIV replication. Brass et al. developed a two-part siRNA screen to detect host factors involved in HIV infection (Brass et al., 2008). With 21,121 pools of siRNA, they identified 273 HIV-dependency factors in TZM-bl cells. Other than 36 host factors, including *CD4*, *CXCR4*, and components of *NF-κB* that were previously implicated in HIV pathogenesis, they revealed the involvement of the Golgi transport proteins Rab6 and Vps53 in HIV entry, TNPO3 in integration, and Med28 in transcription. Another group using a siRNA library composed of 22,329 pools of siRNA targeting 19,709 genes in HeLa P4/R5 cells detected 311 host factors, with an 18-gene overlap with Brass et al. (Zhou et al., 2008). They confirmed the involvement of the SP1/mediator complex and the *NF-κB* signaling pathway in HIV replication. Meanwhile, a study focusing on the early steps of HIV-1 infection revealed >200 genes in human 293T cells that may facilitate HIV infection. Among them, >40 genes specifically regulate the initiation of virus replication (Konig et al., 2008). Although each screen has an overlapping rate of approximately 6%, only three host factors were identified in all three screens, *MED6*, *MED7* (mediator complex), and *RelA* (*NF-κB* complex) (Friedel and Haas, 2011).

Due to the continuous outbreak of seasonal flu and the 2009 pandemic flu, influenza viruses have been vigorously studies using genome-wide RNAi screens. An initial attempt was performed in 2008, covering 90% of the Drosophila genome (Hao et al., 2008). Based on a *Renilla* luciferase reporter gene, >100 host factors were identified that alter influenza replication, including the cytochrome *c* oxidase subunit *COX6A1*, the ATPase *ATP6VoD1*, and the nuclear export factor *NXF1/TAP*. The interferon-inducible trans-membrane proteins IFITM1, 2, and 3 were later discovered to restrict the early stage of influenza A virus replication via a siRNA screen in osteosarcoma cells (U2OS) (Brass et al., 2009). Further tests targeting 19,000 human genes were performed in human lung epithelial A549 cells in 2010 (Konig et al., 2010). Among the 295 host factors identified, 23 factors are necessary for influenza virus entry, and 10 factors are required for post-entry steps. Konig et al. confirm several of the factors using small molecule inhibitors, including that the vATPase and CAMK2B are indeed essential for influenza replication. In addition, a HTS RNAi study using pandemic swine-origin influenza virus identified 168 factors that inhibit virus infection, including the SON DNA binding protein (SON) that controls the trafficking of virions to late endosomes, as well as CDC-like kinase 1 (CLK1). In depth assays further uncovered the role of cyclin-dependent kinase inhibitor 1B (Cdkn1b) in influenza infection by using *p27*^*-*/*-*^ mice (Karlas et al., 2010).

Careful design of RNAi screens also revealed detailed and specific information for poxvirus infection. A library was used to screen 7,000 druggable genes in HeLa cells and clearly shows that Cullin3-based ubiquitination is needed to initiate vaccinia virus (VACV) DNA replication (Mercer et al., 2012). In another study, >500 genes that significantly inhibit, and a similar number that enhance, the replication and spread of VACV were identified from RNAi HTSs with two independent human genome-scale libraries. Functional studies demonstrate that silencing nucleoporin 62 strongly inhibits VACV morphogenesis and has only a modest effect on viral gene expression, thus recapitulating and providing insight into previous studies with enucleated cells (Sivan et al., 2013).

## Conclusions and future perspective

As demonstrated by the various studies described above, the applications of RNAi HTS have been successful for the identification of novel genes that regulated cancer cell growth (either *in vivo* or *in vitro*) and mediated the interactions between viruses and hosts. RNAi HTS has a more broad application as a powerful tool to identify gene networks in a given biological process. Although this approach has been successfully applied in many studies, challenges remain in understanding screen results and, particularly, determining the significance in clinical applications. For example, many studies related to KRAS-driven oncogenic events reveal different major determinants in different cancer cell lines. The non-canonical IκB kinase TBK1 was first identified as a major regulator of mutated KRAS in various cancer cell lines (Barbie et al., 2009). Subsequently, TAK1 and TATA2 have also been found to regulate mutated KRAS in colon cancer cell lines and non-small cell lung cancer cell lines, respectively (Kumar et al., 2012; Singh et al., 2012). However, a set of mitotic regulators including *PLK1* is more important in an isogenic colon cancer cell line (Luo et al., 2009) (Table 2). There are no major overlapping regulators in these studies. This may be caused by investigators using different cell lines with various genetic backgrounds, but these discrepancies may also be caused by unknown reasons.

**Table 2:** RNAi high-throughout screening for genes sensitive to mutated *KRAS*

Group	Cells	Libraries	Characterized genes
Barbie et al. (2009)	19 cell lines with wild-type or mutated *RAS*	Kinase, phosphatase and oncogenes	*TBK1*
Luo et al. (2009)	Isogenic DLD-1	Genome-wide	*PLK1*
Kumar et al. (2012)	28 NSCLC cell lines	7000 human genes	*GATA2*
Singh et al. (2012)	21 mutated *KRAS* colon cancer cell lines	Kinase	*TAK1*

In another example, for the identification of host regulators for HIV replication mentioned as above, overlaps were very limited among the three studies described (Table 1). The largest overlaps were observed between the studies of (Brass et al., 2008) and (Zhou et al., 2008). They use HeLa and HeLa-derived TZM-bl cells and focus on the entire virus life cycle as an interesting biological question. By contrast, König et al. analyzed only the processes subsequent to HIV-1 entry, used different cell lines (293T) (Konig et al., 2008), and show less overlapping results with the other two studies.

Collectively, this leaves one big question: how can one interpret the results from different RNAi HTS experiments? The reasons for the large discrepancies are most likely differences in the experimental setups, such as the cell culture systems, different assays for phenotype detecting, and the various RNAi libraries used in these studies. It will be crucial to carefully define standards for RNAi HTS. The standards will ensure and guide different research groups to generate RNAi HTS datasets as common annotation guidelines for disseminating data online. This will be helpful to compare the datasets generated by different groups and facilitate information sharing.

Also it is difficult, if ever possible to mimic many physiological phenotypes and micro-environments in the cell-based assays. Therefore, *in vivo* RNAi HTS assays as described in mice (Beronja et al., 2013; Fellmann and Lowe, 2014) are likely to become an essential technology advance that facilitates gene function identifications in physiological context. For example, in cancer biology, given the complex interactions of a tumor and its microenvironment, including the communications between tumor cells and surrounding cells (Hanahan and Weinberg, 2011), cell-based *in vitro* RNAi HTS may not able to discover the ideal therapeutic target. However, *in vivo* HTS will provide comprehensive information in much more physiologically relevant conditions, thus should support better design and execution for cancer therapy.

The combination of RNAi HTS and other genomic, transcriptomic, proteomic, and mice modeling techniques will lead to a systemic understanding of gene networks, which are more relevant in a physiological context. In the future, we anticipate that RNAi HTS will be applied as the very first step of many research endeavors, and the results of the screens will provide lead information to design defined validation experiments.

## Abbreviations

EBV: Epstein-Barr virus
dsRNA: double-stranded RNA
HCV: hepatitis C virus
HPV: human papillomaviruses
HTS: high-throughput screening
RISC: RNA-induced silencing complex
RNAi: RNA interference
shRNA: short hairpin RNA
siRNA: short interfering RNA
TNBC: basal-like triple negative breast cancer
